# *APOE* allele frequencies in suspected non-amyloid pathophysiology (SNAP) and the prodromal stages of Alzheimer’s Disease

**DOI:** 10.1371/journal.pone.0188501

**Published:** 2017-11-30

**Authors:** Timothy J. Hohman, Logan Dumitrescu, Amy Oksol, Madison Wagener, Katherine A. Gifford, Angela L. Jefferson, for the Alzheimer’s Disease Neuroimaging Initiative

**Affiliations:** Vanderbilt Memory and Alzheimer’s Center, Department of Neurology, Vanderbilt University Medical Center, Nashville, TN, United States of America; Nathan S Kline Institute, UNITED STATES

## Abstract

Biomarker definitions for preclinical Alzheimer’s disease (AD) have identified individuals with neurodegeneration (ND+) without β-amyloidosis (Aβ-) and labeled them with suspected non-AD pathophysiology (SNAP). We evaluated *Apolipoprotein E* (*APOE*) ε2 and ε4 allele frequencies across biomarker definitions—Aβ-/ND- (n = 268), Aβ+/ND- (n = 236), Aβ-/ND+ or SNAP (n = 78), Aβ+/ND+ (n = 204)—hypothesizing that SNAP would have an *APOE* profile comparable to Aβ-/ND-. Using AD Neuroimaging Initiative data (n = 786, 72±7 years, 48% female), amyloid status (Aβ+ or Aβ-) was defined by cerebrospinal fluid (CSF) Aβ-42 levels, and neurodegeneration status (ND+ or ND-) was defined by hippocampal volume from MRI. Binary logistic regression related biomarker status to *APOE* ε2 and ε4 allele carrier status, adjusting for age, sex, education, and cognitive diagnosis. Compared to the biomarker negative (Aβ-/ND-) participants, higher proportions of ε4 and lower proportions of ε2 carriers were observed among Aβ+/ND- (ε4: OR = 6.23, p<0.001; ε2: OR = 0.53, p = 0.03) and Aβ+/ND+ participants (ε4: OR = 12.07, p<0.001; ε2: OR = 0.29, p = 0.004). SNAP participants were statistically comparable to biomarker negative participants (p-values>0.30). In supplemental analyses, comparable results were observed when coding SNAP using amyloid imaging and when using CSF tau levels. In contrast to *APOE*, a polygenic risk score for AD that excluded *APOE* did not show an association with amyloidosis or neurodegeneration (p-values>0.15), but did show an association with SNAP defined using CSF tau (β = 0.004, p = 0.02). Thus, in a population with low levels of cerebrovascular disease and a lower prevalence of SNAP than the general population, *APOE* and known genetic drivers of AD do not appear to contribute to the neurodegeneration observed in SNAP. Additional work in population based samples is needed to better elucidate the genetic contributors to various etiological drivers of SNAP.

## Introduction

Alzheimer’s disease (AD) is marked by a long preclinical stage in which the pathophysiology has evolved but clinical symptoms are not yet present. The National Institute on Aging and Alzheimer’s Association Workgroup established preclinical AD criteria for research based on biomarkers of β-amyloidosis and neurodegeneration [1]. These criteria led to the subsequent identification of a subset of individuals labeled with suspected non-AD pathophysiology (SNAP) due to biomarker evidence of neurodegeneration without β-amyloidosis. SNAP does not align with the leading theoretical progression of dynamic biomarkers during preclinical AD in which β-amyloidosis evidence appears first, followed by neurodegeneration [2] Among cognitively normal older adults, both SNAP and amyloid positive profiles are associated with worse cognitive trajectories when compared to biomarker negative individuals, but the fastest rate of decline is observed in individuals who are positive for markers of both amyloid and neurodegeneration [3]. Among individuals with mild cognitive impairment (MCI), a prodromal stage of dementia, SNAP is associated with increased risk of progressive cognitive decline [4], smaller cortical volume [5], and higher burden of white matter hyperintensities [5] compared to biomarker negative peers. In contrast, when compared to individuals with evidence of both β-amyloidosis and neurodegeneration, SNAP appears to be less likely to progress to clinical AD [6, 7]. Moreover, there is inconsistent evidence of clinical progression among SNAP individuals who are cognitively normal at baseline, with some studies reporting very low conversion rates [6, 8].

Recent genetic exploration of these biomarker groups suggests the frequency of the *APOE* ε4 genotype (a genetic susceptibility risk factor for AD) may be lower in SNAP when compared to amyloid positive or amyloid positive/neurodegeneration positive groups [4]. This finding suggests that the neurodegeneration and cognitive decline observed in SNAP may have a unique etiology that is not linked to *APOE*. In addition to the ε4 effect, it is well known that the *APOE* ε2 allele is protective against AD [9]. Such a protective role may be due to lower levels of AD neuropathology among carriers of the ε2 allele [10], although work in the oldest old has suggested ε2 is associated with preserved cognition but high levels of neuropathology [11]. Additionally, studies of the possible underlying neuropathologies of SNAP, including tangle predominant dementia [12], cerebrovascular disease [13], and argyrophilic grain disease [14], have highlighted an increase in disease risk among *APOE* ε2 allele carriers. Biomarker groups based on the preclinical AD criteria thus provide a tremendous opportunity to better understand the association between *APOE* and biomarkers of AD neuropathology and neurodegeneration.

This study assesses the frequency of all *APOE* alleles across biomarker groups among individuals diagnosed with normal cognition and MCI. We hypothesize that the *APOE* effect will be specific to the AD cascade [15], such that when compared to biomarker negative participants, we will observe higher frequencies of the *APOE* ε4 allele and lower frequencies of the ε2 allele among the amyloid positive participants and among the biomarker (both amyloid and neurodegeneration) positive participants but not among the SNAP participants.

## Materials and methods

Participants were selected from the AD Neuroimaging Initiative (ADNI). ADNI launched in 2003 as a public-private partnership. The original ADNI study enrolled approximately 800 participants, aged 55–90 years, excluding serious neurological disease (other than AD), history of brain lesion or head trauma, and history of psychoactive medication use (for full inclusion/exclusion criteria see http://www.adni-info.org). Informed written consent was obtained from all participants at each site, and analysis of ADNI’s publically available database was approved by our local Institutional Review Board prior to data analysis.

### Participants

Data were accessed on 10/10/2017. ADNI participants were selected for this study if they had cerebrospinal fluid (CSF) biomarker data, an MRI measure of hippocampal volume, and *APOE* genotype data available for analysis. Participants were excluded if there MRI visit and CSF visit were more than 6 months apart. These criteria resulted in 786 participants, including 305 with NC and 481 with MCI. Participants with AD were excluded.

### *APOE* genotyping

*APOE* genotyping in ADNI has been outlined previously [16]. Briefly, PCR was followed by HhaI restriction enzyme digestion and results were visualized using ethidium bromide staining.

### Hippocampal volume

The ADNI brain MRI protocol has been reported in detail [17]. The current study included 1.5T T1-weighted structural imaging data from ADNI-1 and 3T data from ADNI-2/GO. Cortical reconstruction and volumetric segmentation were performed with the FreeSurfer image analysis suite version 4.3 in ADNI-1 and 5.1 in ADNI-2/GO (http://surfer.nmr.mgh.harvard.edu/; [18–20]. FreeSurfer processing in ADNI, including quality control (QC) procedures, have been described elsewhere [21]. We only included participants who passed QC procedures. We used left hippocampal volume, right hippocampal volume, and estimated intracranial volume (ICV) as defined by FreeSurfer [22].

### Biomarker groups

We used CSF amyloid positivity and MRI hippocampal volume neurodegeneration (ND) to create four biomarker groups: Biomarker Negative (Aβ-/ND-), Amyloid Positive (Aβ+/ND-), Neurodegeneration Positive (or SNAP; Aβ-/ND+), and Biomarker Positive (Aβ+/ND+).

Amyloid positivity was based on the assay-specific cut-point of Aβ-42≤192 pg/mL [23]. CSF protocols, including the quantification of Aβ-42, have been detailed previously for ADNI [23, 24].

Classification as ND+ requires an adjusted hippocampal volume≤6723 mm^3^, based on previously published criteria [3]. Briefly, adjusted hippocampal volume (*adj*.*HV*) was calculated using the following formula:

adj.HV=total.HV−(a*[ICV−mean.ICV.norm])

where *total*.*HV* is the added values of right and left hippocampal volume, *a* is the regression coefficient when *total*.*HV* is regressed against ICV in the NC group, and *mean*.*ICV*.*norm* is the mean ICV for all NC participants.

### Statistical analyses

All statistical analyses were performed using R Studio (version 0.99.485, www.rstudio.com). NC and MCI group comparisons were completed using χ^2^ for categorical variables and t-tests for continuous variables.

Unadjusted differences in *APOE* genotype frequencies were evaluated across all biomarker groups using χ^2^. Next, two binary logistic regression models assessed differences between the Aβ-/ND- group and the three remaining biomarker groups (i.e., Aβ+/ND-, Aβ-/ND+ [SNAP], and Aβ+/ND+), adjusting for age, sex, education, and cognitive diagnosis. In the first logistic regression model, *APOE* ε4 carrier status was set as the binary outcome (carriers = ε2/ε4, ε3/ε4, ε4/ε4 versus non-carriers = ε2/ε2, ε2/ε3, ε3/ε3). In the second logistic regression analysis, *APOE* ε2 carrier status was set as the binary outcome (carriers = ε2/ε2, ε2/ε3, ε2/ε4 versus non-carriers = ε3/ε3, ε3/ε4, ε4/ε4). Finally, using two separate χ^2^ tests, post-hoc pairwise comparisons restricted to the groups carrying at least one AD biomarker evaluated differences in *APOE* frequency between SNAP and each of the other two biomarker positive groups (Aβ+/ND- and Aβ+/ND+). In all analyses, significance was set *a priori* as α = 0.05. Correction for multiple comparisons was performed using the Bonferroni procedure for the 6 planned statistical comparisons from our primary analyses (corrected-α = 0.008).

Supplemental analyses were run to test the impact of diagnostic status on our results and to evaluate differences when defining SNAP based on CSF tau levels rather than hippocampal volume. First, we evaluated biomarker group x diagnosis interactions on *APOE* ε2 carrier status and ε4 carrier status separately using the same logistic regression models outlined above. Second, we re-ran all analyses stratified by diagnosis, including logistic models and post-hoc χ^2^ tests. Third, we re-ran primary analyses removing individuals with the ε2/ε4 genotype to ensure these individuals were not driving group results. Fourth, we re-ran primary analyses using CSF tau biomarker levels based on a previously established cut-point (CSF tau≥93 pg/mL; [23]. Fifth, we repeated primary analyses covarying for white-matter hyperintensity burden quantified from 3D axial T2-weighted fluid-attenuated inversion recovery images collected as part of the ADNI-2 protocol and quantified using an established pipeline that has been reported in detail elsewhere [25]. Total white-matter hyperintensity volume was entered into logistic models as a continuous covariate, and we evaluated whether effects persisted when including this covariate. Finally, given potential concerns over batch effects for CSF and scanner effects for MRI, we chose to recalculate all biomarker groups using previously processed PET florbetapir data [26] to define amyloid positivity and restricted MRI data to 3T images from ADNI2/GO.

Finally, additional analyses were run leveraging a polygenic risk score (PGRS) of AD based on the top 21 loci from the Lambert et al. meta-analysis [27]. All ADNI genotype data were imputed to the HRC reference panel and genetic processing was performed using PLINK (version 1.9) [28]. Quality control steps excluded SNPs with a minor allele frequency < 1%, with a genotyping rate < 99%, and out of Hardy Weinberg equilibrium (p<0.000001). Risk scores were calculated using the score function in PLINK. The association between PGRS was regressed on age, sex, diagnostic group, and biomarker group. One analysis was run using biomarker group defined using neurodegeneration definitions, and one analysis was run using CSF tau definitions.

## Results

Demographic and clinical characteristics are presented in **Table 1**. The distribution of biomarker groups differed between NC and MCI participants (χ^2^(3) = 104, p<0.001). While the frequency of Aβ+/ND- (33% in NC, 28% in MCI) and SNAP (10% in both NC and MCI) were comparable between groups, the NC group showed a higher frequency of Aβ-/ND- (50% in NC, 24% in MCI) and a lower frequency of Aβ+/ND+ (8% in NC, 38% in MCI).

**Table 1:** Sample characteristics.

	Clinical Diagnosis		Statistical Test
	Normal Cognition	Mild Cognitive Impairment	
Sample Size, n	305	481	
Age, years	73±6	72±7	t(520.5) = -3.88, p<0.001
Sex, % female	53%	43%	χ^2^(1) = 7.39, p = 0.007
Education, years	16±3	16±3	t(520.5) = -1.29, p = 0.196
CSF Aβ-42, pg/mL	201±52	172±52	t(520.5) = -7.69, p<0.001
CSF Total Tau, pg/mL	67±30	92±58	t(520.5) = 7.89, p<0.001
Hippocampus Volume, mm^3^	7482±787	6769±1172	t(520.5) = -10.19, p<0.001
Composite Memory Performance, z-score	1.06±0.55	0.20±0.69	t(520.5) = -19.35, p<0.001
Composite Executive Function Performance, z-score	0.81±0.72	0.26±0.80	t(520.5) = -9.94, p<0.001
PET Amyloid Burden, SUVR^#^	1.12±0.18	1.22±0.23	t(520.5) = 5.81, p<0.001
Biomarker Group, n (%)			χ^2^(3) = 104.19, p<0.001
Aβ-/ND-	153 (50%)	115 (24%)	
Aβ+/ND-	100 (33%)	136 (28%)	
Aβ-/ND+ (SNAP)	29 (10%)	49 (10%)	
Aβ+/ND+	23 (8%)	181 (38%)	
*APOE* Genotype, n (%)			χ^2^(5) = 52.02, p<0.001
ε2/ε2	0 (0%)	1 (0.2%)	
ε2/ε3	43 (14%)	27 (6%)	
ε2/ε4	4 (1%)	7 (1%)	
ε3/ε3	178 (58%)	212 (44%)	
ε3/ε4	73 (24%)	184 (38%)	
ε4/ε4	7 (2%)	50 (10%)	

Results are presented as mean ± standard deviation unless otherwise indicated. Hippocampal volume adjusted for intracranial volume.

^#^PET Amyloid Imaging present at same visit for 212 NC and 339 MCI participants.

**Fig 1:**
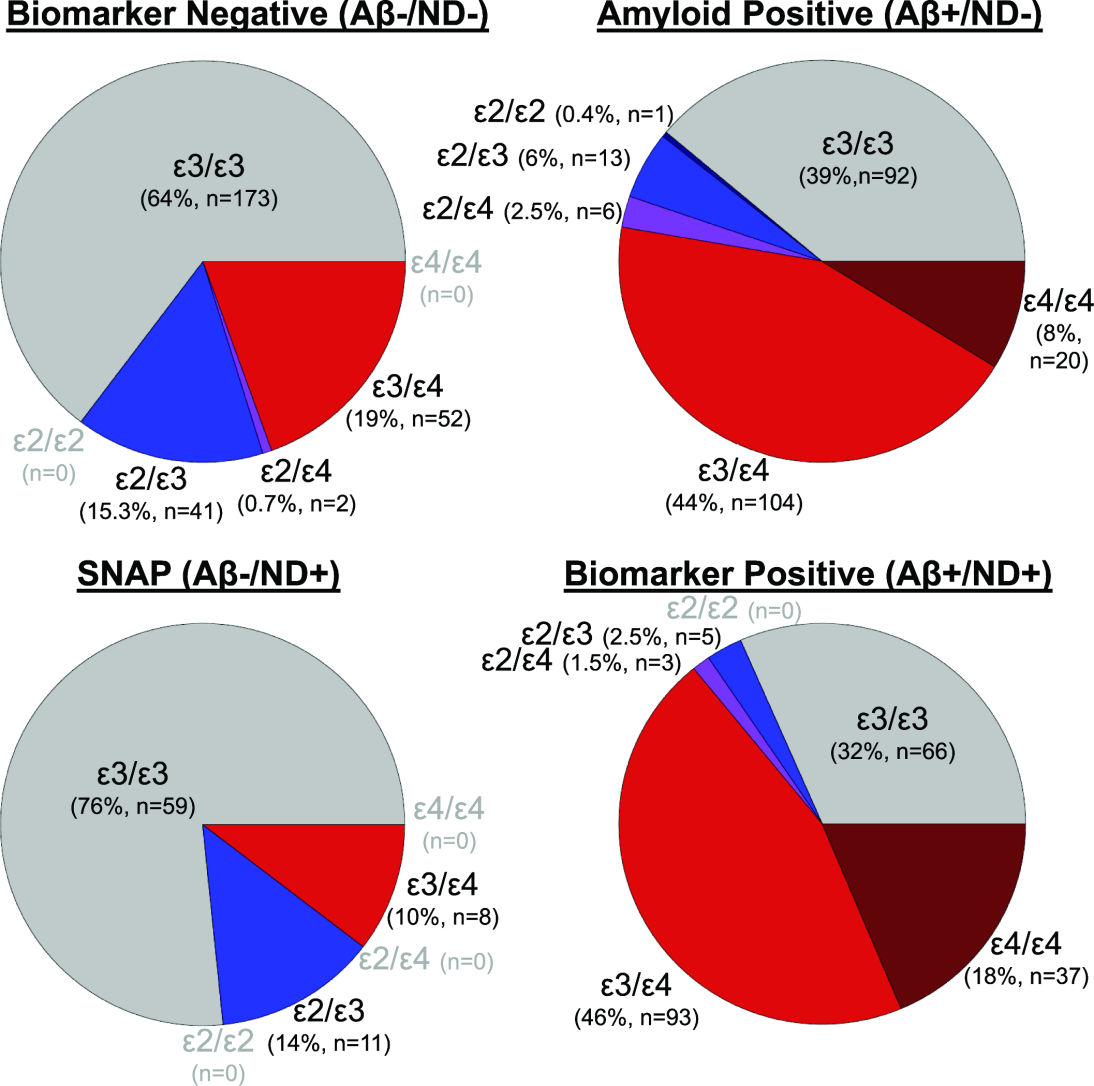
APOE genotypes across AD biomarker. Pie charts are presented by biomarker group based on amyloid status defined using levels of cerebrospinal fluid amyloid-β 42 (Aβ) and neurodegeneration defined using hippocampal volume (ND). Colors represent APOE genotype whereby gray represents homozygous ε3 allele carriers, shades of red represent ε4 allele carriers, shades of blue represent ε2 allele carriers, and purple is used to represent ε2/ε4 carriers. Sample sizes are presented below the segment label for each allele combination. Allele combinations that do not have any participants within a given biomarker group are labeled in light grey font.

There was an unadjusted difference in the distribution of *APOE* genotypes across biomarker groups (χ^2^(15) = 179, p<0.001, see **Fig 1**), which appears secondary to group differences in both ε4 (χ^2^(3) = 147, p<0.001) and ε2 allele frequencies (χ^2^(3) = 20, p<0.001). In fully adjusted models, the Aβ+/ND- and Aβ+/ND+ groups presented less frequently with the *APOE* ε2 allele compared to the Aβ-/ND- group (p-values<0.03), whereas the ε2 frequency was similar between SNAP and Aβ-/ND- (p = 0.97). Only the difference in ε2 frequency between Aβ+/ND+ and Aβ-/ND- survived correction for multiple comparisons. Similarly, the Aβ+/ND- and Aβ+/ND+ groups presented more frequently with the ε4 allele when compared to the Aβ-/ND- group (p-values<0.001) and survived correction for multiple comparisons, whereas the SNAP and Aβ-/ND- groups were similar in frequency for the ε4 allele (p = 0.30). See **Table 2** for details.

**Table 2:** Associations between biomarker groups and APOE carrier status.

	Odds Ratio (95% CI)	p-value
***APOE ε2***		
Age	0.99 (0.96–1.03)	0.809
Sex (female)	1.04 (0.65–1.67)	0.864
Cognitive Diagnosis (MCI)	**0.57 (0.34–0.95)**	**0.031**
Aβ+/ND-*	**0.53 (0.29–0.92)**	**0.028**
Aβ-/ND+ (SNAP)*	0.99 (0.45–2.04)	0.972
Aβ+/ND+*	**0.29 (0.12–0.65)**	**0.004**
***APOE ε4***		
Age	**0.91 (0.89–0.94)**	**<0.001**
Sex (female)	0.93 (0.66–1.30)	0.666
Cognitive Diagnosis (MCI)	1.36 (0.93–1.98)	0.111
Aβ+/ND-*	**6.23 (4.09–9.64)**	**<0.001**
Aβ-/ND+ (SNAP)*	0.65 (0.27–1.41)	0.301
Aβ+/ND+*	**12.07(7.25–20.53)**	**<0.001**

**Boldface** signifies effects that are significant at p<0.05.

^*^ 4 level categorical variable for biomarker group with Aβ-/ND- set as the referent

Pairwise comparisons between the biomarker-positive groups (i.e., Aβ+/ND-, SNAP, and Aβ+/ND+) suggest only the SNAP group differed from the Aβ+/ND+ group for the ε2 allele (p = 0.005). No other pairwise comparison for the ε2 allele reached statistical significance (p-values>0.08). Regarding ε4 allele frequency, all groups differed from one another (p-values<0.03). See **Table A in S1 Supplemental Materials** for details.

Supplemental analyses evaluating diagnostic interactions are presented in **Table B in S1 Supplemental Materials.** We did not observe an interaction between biomarker group and diagnosis on *APOE* ε4 (p = 0.17), or on ε2 status (p = 0.09). Stratified models are presented in **Table C** and **D in S1 Supplemental Materials.**

Supplemental analyses of pairwise comparisons stratified by diagnosis are presented in **Table E** and **F in S1 Supplemental Materials**. While ε4 results appeared to be consistent across diagnostic categories, the significant difference between the *Aβ+/ND+* positive group and SNAP in the frequency of ε2 was only present among cognitively normal individuals (p = 0.02).

Supplemental analyses using biomarker groups defined with CSF tau are presented in **Table G in S1 Supplemental Materials**. The results are consistent with the reported findings when using groups defined based on hippocampal volume.

Supplemental analyses removing ε2/ε4 carriers are presented in **Table H in S1 Supplemental Materials**. Removing these individuals did not change the pattern of results.

Supplemental analyses including white-matter hyperintensity burden as a covariate are presented in **Table I in S1 Supplemental Materials**. Although the sample size is reduced due to the fact that FLAIR images were only acquired as part of the ADNI-2 protocol, the pattern of results remain largely consistent with the unadjusted reports above, suggesting differences in white-matter hyperintensity burden are not contributing to the observed associations between *APOE* and biomarker groups.

Supplemental analyses leveraging amyloid PET data to define amyloid positivity in biomarker groups are presented in **Table J in S1 Supplemental Materials**. Again, the pattern of results largely recapitulated the CSF biomarker results, including strong associations between amyloid groups and *APOE* genotypes.

Results evaluating PGRS associations with biomarker groups are presented in **Table K in S1 Supplemental Materials**. The PGRS for AD was unrelated to amyloidosis or neurodegeneration, but did show an association with SNAP defined using CSF tau (F(3,518) = 2.76, p = 0.04) whereby the Aβ-42-/Tau+ group (p = 0.02) and the combined Aβ-42+/Tau+ group (p = 0.04) had higher PGRS than the biomarker negative group.

Finally, demographic and clinical characteristics of the sample are presented in **Table 3** to provide a more thorough overview of these biomarker groups.

**Table 3:** Descriptive statistics by biomarker group.

	Biomarker Group				Statistical Test
	Aβ-/ND-	Aβ+/ND-	Aβ-/ND+	Aβ+/ND+	
Sample Size, n	268	236	78	204	
Age, years	70.11±6.56	71.68±6.64	75.15±6.64	75.12±6.09	F(3,782) = 29, p<0.001^a^^,^^b^^,^^c^^,^^d^^,^^e^
Sex, % female	50%	51%	38%	42%	χ^2^(3) = 6.81, p = 0.078
Education, years	16.26±2.59	16.16±2.66	16.45±2.91	15.94±2.93	F(3,782) = 1, p = 0.458
CSF Aβ-42, pg/mL	236.82±26.69	146.87±25.04	235.64±27.25	135.00±25.10	F(3,782) = 883, p<0.001^a^^,^^c^^,^^d^^,^^e^^,^^f^
CSF Total Tau, pg/mL	57.66±22.82	90.56±49.61	62.13±25.34	112.19±64.42	F(3,759) = 62, p<0.001^a^^,^^c^^,^^d^^,^^e^^,^^f^
Hippocampus Volume, mm^3^	7835±651	7559±610	5887±699	5858±636	F(3,782) = 505, p<0.001^a^^,^^b^^,^^c^^,^^d^^,^^e^
Composite Memory Performance, z-score	0.92±0.62	0.63±0.68	0.51±0.77	-0.07±0.66	F(3,782) = 88, p<0.001^a^^,^^b^^,^^c^^,^^e^^,^^f^
Composite Executive Function Performance, z-score	0.82±0.77	0.49±0.76	0.45±0.74	0.00±0.72	F(3,782) = 45, p<0.001^a^^,^^b^^,^^c^^,^^e^^,^^f^
PET Amyloid Burden, SUVR^#^	1.02±0.07	1.27±0.19	1.02±0.10	1.40±0.22	F(3,547) = 172, p<0.001^a^^,^^c^^,^^d^^,^^e^^,^^f^

Significant pairwise statistical comparisons are indicated in the following manner

^a^Aβ-/ND- v. Aβ+/ND-

^b^Aβ-/ND- v. Aβ-/ND+

^c^Aβ-/ND- v. Aβ+/ND+

^d^Aβ+/ND- v. Aβ-/ND+

^e^Aβ+/ND- v. Aβ+/ND+

^f^Aβ-/ND+ v. Aβ+/ND+.

Results are presented as mean ± standard deviation unless otherwise indicated. Hippocampal volume adjusted for intracranial volume.

^#^PET Amyloid Imaging present at same visit for a subset of participants.

## Discussion

We evaluated the distribution of *APOE* allele frequencies in biomarker groups operationally defined using the classification scheme for preclinical AD. As expected, we observed a lower ε2 allele frequency and a higher ε4 allele frequency in participants with evidence of amyloidosis (i.e., Aβ+/ND- and Aβ+/ND+) compared to biomarker negative (Aβ-/ND-) participants. As hypothesized, we observed comparable *APOE* allele frequencies among participants with SNAP compared to participants who were biomarker negative (Aβ-/ND-). These results add to growing evidence that SNAP is not driven by an *APOE* pathway and that the neurodegeneration that defines SNAP likely has a unique etiology that differs from AD.

To our knowledge, our results are among the first to report the frequency of the *APOE* ε2 allele in biomarker groups used to define preclinical AD. We observed lower ε2 frequencies in the amyloidosis (Aβ+/ND- and Aβ+/ND+) groups compared to the biomarker negative (Aβ-/ND-) group, but we did not observe an ε2 frequency difference between the SNAP (Aβ-/ND+) and biomarker negative (Aβ-/ND-) groups. In post-hoc pairwise comparisons, we observed a higher ε2 frequency in SNAP compared the biomarker positive (Aβ+/ND+) groups. As highlighted in the introduction, the *APOE* ε2 allele is particularly interesting to evaluate in the context of SNAP because it has a well-documented protective effect on AD pathology [10], yet it has been associated with an *increased* risk of cerebrovascular disease [13]. Similarly, an increased frequency of *APOE* ε2 have been reported in argyrophilic grain disease [14] and hemorrhage in cerebral amyloid angiopathy [29], suggesting *APOE* ε2 may play a unique role in the non-AD pathological processes implicated in SNAP [4]. However, our findings suggest that, like the ε4 allele, the frequency of the ε2 allele is comparable to observations in biomarker negative (Aβ-/ND-) individuals. Thus, while ε2 may protect against the AD cascade in SNAP, the similar frequency distribution in biomarker negative (Aβ-/ND-) and SNAP groups suggests ε2 is unlikely to contribute to the neuropathology underlying SNAP. That said, the low ε2 frequency in SNAP emphasizes the need for further exploration in larger datasets where subtle differences in ε2 between SNAP and biomarker negative participants may be more readily detectable.

We also observed expected differences in *APOE* ε4 frequency among biomarker groups consistent with previous work in both cognitively normal [3] and MCI [4] cohorts, suggesting the ε4 allele frequency is lower in participants with SNAP compared to amyloidosis (Aβ+/ND- or Aβ+/ND+[2, 30] The *APOE* ε4 allele is thought to promote risk for AD through an amyloid rather than tau pathway [31], and the present results suggest that *APOE* ε4 does not exert a major effect in the pathophysiology of neurodegeneration in SNAP. It is interesting to note that one of the primary hypothesized pathways of neurodegeneration in SNAP, hippocampal sclerosis (HS), also shows no association with *APOE* when AD neuropathology is not present in the brain [32]. Similarly, primary age-related tauopathy (PART) appears unrelated to *APOE* [33, 34], although debate surrounds this issue [35]. The present results suggest that neither *APOE* ε2 nor ε4 alleles correspond to the neurodegeneration or tauopathy observed in SNAP. In contrast to PART and HS, there is some evidence of an association between arteriolosclerosis and *APOE* in the presence of amyloidosis [36], and some evidence of an inverse association between *APOE ε2* and markers of cerebrovascular disease relative to what is observed in AD [13]. Thus, there is a need in the field to more carefully characterize the underlying neuropathology of SNAP across cohorts to more fully elucidate risk and protective factors. The ADNI participant screening procedures included an exclusion for overt cerebrovascular disease, suggesting it may not be ideally suited to evaluate the contributions of genetic drivers of cerebrovascular disease within SNAP. This feature of the ADNI cohort leaves open the possibility that *APOE* may contribute to SNAP when the driven by a cerebrovascular etiology.

In supplemental analyses, we observed an association between SNAP groups defined using CSF tau levels and PGRS for AD, suggesting that while *APOE* does not appear related to SNAP, some of the genetic risk associated with clinical AD is relevant to tau even in the absence of amyloidosis (see **Table K in S1 Supplemental Materials**). These findings recapitulate the lack of association between CSF amyloid, baseline hippocampal volume, and PGRS in a previous ADNI publication [37], although CSF tau was not evaluated in that manuscript. The patterns of association between PGRS and biomarker groups (that differs from the *APOE* association with biomarker groups) highlights the need to better understand the genetic architecture of the neuropathological features of AD, and characterize how genetic factors differentially drive the neuropathological and clinical progression in AD. The amount genetic overlap between AD and SNAP likely depends on the various etiologies that underlie SNAP. Regardless of etiology, however, we provide strong evidence that *APOE* is not associated with SNAP.

Previous reports of these biomarker groups in ADNI, both among individuals with normal cognition at baseline [38] and among individuals with MCI at baseline [4], are consistent with the clinical and cognitive group differences we observed here (**Table 3**). Specifically, we observed expected group differences in cognition, biomarker status, and hippocampal volume including marked differences in cognition and hippocampal volume among SNAP individuals compared to biomarker negative individuals. Given that some of the group differences between SNAP and biomarker negative individuals depend on the way SNAP is defined (e.g., CSF tau levels), it appears that moving toward a multi-biomarker diagnostic scheme accounting for amyloid, tau, and neurodegeneration (e.g., the A/T/N classification [39]) will more accurately characterize the neuropathology underlying SNAP. Unfortunately, we did not have a large enough sample size to fully evaluate *APOE* differences among the A/T/N groups. Larger sample sizes with biomarker and imaging data are needed to fully characterize the genetic etiology of these neuropathological processes.

These findings must be interpreted in the context of several known limitations of the ADNI dataset. The frequency of SNAP observed in the present analysis (10%) is notably lower than some previous reports of SNAP (25%)[3]. This discrepancy may be due in part to the fact that, as previously mentioned, ADNI underrepresents the burden of cerebrovascular disease among older adults given sample recruitment criteria (Hachinski score<4) and MRI contraindications. Importantly, SNAP is a particularly strong driver of MCI in population-based studies more accurately reflecting the mixed etiologies of age-related cognitive impairment. Thus, it will be critical to further assess the association between *APOE* and SNAP in more representative cohorts. There is also an over-representation of *APOE* ε4 carriers in ADNI, highlighting the need to further investigate the full breadth of *APOE* alleles in additional samples that better represent the frequency of SNAP and each *APOE* allele. The ADNI cohort is also enriched for White, highly educated individuals who do not reflect the general population of older adults. Larger and more representative samples are required to fully evaluate the role of *APOE* ε2 in SNAP and will likely require a meta-analysis across multiple datasets. Therefore, it is imperative that future SNAP studies report the full spectrum of *APOE* genotype to more easily facilitate cross-study evaluations of possible genetic effects. As previously mentioned, there is growing emphasis on biomarker definitions that combine a marker of amyloid, a marker of tau, and a marker of neurodegeneration, called the A/T/N scheme [39]. Additional work focusing on *APOE* genotype in these smaller sub-groups will require much larger sample sizes, but the present results leave open the possibility that ε2 may be involved in neuropathological processes that underlie a more specific non-amyloid sub group (A-/T+/N+, A-/T+/N-, or A-/T-/N+).

In conclusion, this study is among the first to provide evidence that the *APOE* ε2 allele frequency is comparable between SNAP and biomarker negative individuals and adds to existing evidence [4] that the frequency of the *APOE* ε4 allele is lower in participants with SNAP. These results collectively suggest that any genetic etiology of neurodegeneration in SNAP is likely through a non-*APOE* pathway, further supporting the notion that SNAP represents a heterogeneous subset of older adults experiencing non-AD neurodegeneration.

## Supporting information

S1 Supplemental Materials (DOCX)
