# Supplementary material for: APOE allele frequencies in suspected non-amyloid pathophysiology (SNAP) and the prodromal stages of Alzheimer’s Disease
Source: PLoS One. 2017 Nov 30;12(11):e0188501. doi: 10.1371/journal.pone.0188501 (PMC5708777; doi:10.1371/journal.pone.0188501)
Supplement: S1 Supplemental Materials — (DOCX) [file pone.0188501.s001.docx]

## Table A: Pairwise Comparisons for APOE Analysis

| **Outcome: *APOE* Carrier Status** | **χ^2^** | **p-value** |
| --- | --- | --- |
| ***APOE ε2*** |  |  |
| Aß+/ND- vs. Aß-/ND+ (SNAP) | 1.50 | 0.220 |
| Aß+/ND+ vs Aß-/ND+ (SNAP) | **7.76** | **0.005** |
| Aß+/ND- vs. Aß+/ND+ | 3.08 | 0.079 |
| ***APOE ε4*** |  |  |
| Aß+/ND- vs. Aß-/ND+ (SNAP) | **43.57** | **<0.001** |
| Aß+/ND+ vs Aß-/ND+ (SNAP) | **64.42** | **<0.001** |
| Aß+/ND- vs. Aß+/ND+ | **4.70** | **0.030** |

**Boldface** signifies effects that are significant at p<0.05.

Table B: Interaction between Biomarker Groups and Clinical Diagnosis on APOE Carrier Status

|  | **Degrees of Freedom** | **χ^2^** | **p-value** |
| --- | --- | --- | --- |
| ***APOE ε2*** |  |  |  |
| Age | 1 | 0.42 | 0.515 |
| Sex (female) | 1 | 0.20 | 0.659 |
| Cognitive Diagnosis (MCI) | **1** | **13.63** | **<0.001** |
| Biomarker Group | **3** | **12.61** | **0.006** |
| Biomarker Group x Diagnosis | 3 | 6.42 | 0.093 |
| ***APOE ε4*** |  |  |  |
| Age | **1** | **18.17** | **<0.001** |
| Sex (female) | 1 | 0.18 | 0.669 |
| Cognitive Diagnosis (MCI) | **1** | **34.27** | **<0.001** |
| Biomarker Group | **3** | **159.98** | **<0.001** |
| Biomarker Group x Diagnosis | 3 | 5.01 | 0.171 |

**Boldface** signifies effects that are significant at p<0.05.

Table C: Associations between Biomarker Groups and APOE Carrier Status among Individuals with Normal Cognition

|  | **Odds Ratio (95% CI)** | **p-value** |
| --- | --- | --- |
| ***APOE ε2*** |  |  |
| Age | 0.99 (0.93-1.05) | 0.668 |
| Sex (female) | 1.36 (0.72-2.65) | 0.350 |
| Aβ+/ND-^*^ | 0.54 (0.24-1.13) | 0.113 |
| Aβ-/ND+ (SNAP)^*^ | 1.75 (0.66-4.38) | 0.241 |
| Aβ+/ND+^*^ | 0.00 (0.00-0.00) | 0.984 |
| ***APOE ε4*** |  |  |
| Age | **0.91 (0.86-0.96)** | **<0.001** |
| Sex (female) | 0.98 (0.56-1.72) | 0.944 |
| Aβ+/ND-^*^ | **4.23 (2.33-7.86)** | **<0.001** |
| Aβ-/ND+ (SNAP)^*^ | 0.22 (0.01-1.12) | 0.145 |
| Aβ+/ND+^*^ | **7.90 (2.85-22.56)** | **<0.001** |

**Boldface** signifies effects that are significant at p<0.05.

**^*^** 4-level categorical variable for biomarker group with Aβ-/ND- set as the referent

Table D: Associations between Biomarker Groups and APOE Carrier Status among Individuals with Mild Cognitive Impairment

|  | **Odds Ratio (95% CI)** | **p-value** |
| --- | --- | --- |
| ***APOE ε2*** |  |  |
| Age | 1.01 (0.95-1.06) | 0.807 |
| Sex (female) | 0.70 (0.33-1.43) | 0.330 |
| Aβ+/ND-^*^ | 0.45 (0.18-1.07) | 0.078 |
| Aβ-/ND+ (SNAP)^*^ | 0.38 (0.08-1.33) | 0.163 |
| Aβ+/ND+^*^ | **0.28 (0.10-0.74)** | **0.012** |
| ***APOE ε4*** |  |  |
| Age | **0.91 (0.88-0.94)** | **<0.001** |
| Sex (female) | 0.93 (0.60-1.42) | 0.731 |
| Aβ+/ND-^*^ | **9.15 (5.02-17.25)** | **<0.001** |
| Aβ-/ND+ (SNAP)^*^ | 1.08 (0.39-2.73) | 0.880 |
| Aβ+/ND+^*^ | **16.61 (8.75-32.89)** | **<0.001** |

**Boldface** signifies effects that are significant at p<0.05.

**^*^** 4-level categorical variable for biomarker group with Aβ-/ND- set as the referent

## Table E: Pairwise Comparisons for APOE Analysis among Individuals with Normal Cognition

| **Outcome: *APOE* Carrier Status** | **χ^2^** | **p-value** |
| --- | --- | --- |
| ***APOE ε2*** |  |  |
| Aß+/ND- vs. Aß-/ND+ (SNAP) | 3.69 | 0.055 |
| Aß+/ND+ vs Aß-/ND+ (SNAP) | **5.53** | **0.019** |
| Aß+/ND- vs. Aß+/ND+ | 1.59 | 0.207 |
| ***APOE ε4*** |  |  |
| Aß+/ND- vs. Aß-/ND+ (SNAP) | **13.81** | **<0.001** |
| Aß+/ND+ vs Aß-/ND+ (SNAP) | **11.84** | **<0.001** |
| Aß+/ND- vs. Aß+/ND+ | 0.04 | 0.842 |

**Boldface** signifies effects that are significant at p<0.05.

## Table F: Pairwise Comparisons for APOE Analysis among Individuals with Mild Cognitive Impairment

| **Outcome: *APOE* Carrier Status** | **χ^2^** | **p-value** |
| --- | --- | --- |
| ***APOE ε2*** |  |  |
| Aß+/ND- vs. Aß-/ND+ (SNAP) | 0.00 | 1.000 |
| Aß+/ND+ vs Aß-/ND+ (SNAP) | 0.01 | 0.906 |
| Aß+/ND- vs. Aß+/ND+ | 0.37 | 0.543 |
| ***APOE ε4*** |  |  |
| Aß+/ND- vs. Aß-/ND+ (SNAP) | **30.83** | **<0.001** |
| Aß+/ND+ vs Aß-/ND+ (SNAP) | **40.89** | **<0.001** |
| Aß+/ND- vs. Aß+/ND+ | 0.55 | 0.458 |

**Boldface** signifies effects that are significant at p<0.05.

Table G: Associations between Biomarker Groups Defined with CSF Tau Levels and APOE Carrier Status

|  | **Odds Ratio (95% CI)** | **p-value** |
| --- | --- | --- |
| ***APOE ε2*** |  |  |
| Age | 0.99 (0.96-1.03) | 0.680 |
| Sex (female) | 1.07 (0.66-1.73) | 0.779 |
| Cognitive Diagnosis (MCI) | **0.55 (0.33-0.90)** | **0.018** |
| Aβ+/Tau-^*^ | **0.53 (0.30-0.92)** | **0.028** |
| Aβ-/Tau+ (SNAP)^*^ | 0.54 (0.12-1.63) | 0.331 |
| Aβ+/Tau+^*^ | **0.28 (0.12-0.59)** | **0.002** |
| ***APOE ε4*** |  |  |
| Age | **0.92 (0.89-0.94)** | **<0.001** |
| Sex (female) | 0.92 (0.66-1.30) | 0.654 |
| Cognitive Diagnosis (MCI) | **1.47 (1.02-2.12)** | **0.039** |
| Aβ+/Tau-^*^ | **6.70 (4.41-10.34)** | **<0.001** |
| Aβ-/Tau+ (SNAP)^*^ | 1.33 (0.42-3.55) | 0.591 |
| Aβ+/Tau+^*^ | **12.86 (8.05-20.98)** | **<0.001** |

**Boldface** signifies effects that are significant at p<0.05.

**^*^** 4 level categorical variable for biomarker group with Aβ-/Tau- set as the referent

Table H: Associations between Biomarker Groups and APOE Carrier Status After Removing ε2/ ε4 Carriers

|  | **Odds Ratio (95% CI)** | **p-value** |
| --- | --- | --- |
| ***APOE ε2*** |  |  |
| Age | 1.00 (0.96-1.04) | 0.917 |
| Sex (female) | 0.96 (0.58-1.59) | 0.873 |
| Cognitive Diagnosis (MCI) | **0.53 (0.30-0.92)** | **0.025** |
| Aβ+/ND-^*^ | **0.38 (0.20-0.72)** | **0.004** |
| Aβ-/ND+ (SNAP)^*^ | 1.01 (0.45-2.10) | 0.989 |
| Aβ+/ND+^*^ | **0.19 (0.06-0.48)** | **0.001** |
| ***APOE ε4*** |  |  |
| Age | **0.91 (0.89-0.94)** | **<0.001** |
| Sex (female) | 0.92 (0.66-1.29) | 0.634 |
| Cognitive Diagnosis (MCI) | 1.40 (0.95-2.04) | 0.087 |
| Aβ+/ND-^*^ | **6.19 (4.04-9.64)** | **<0.001** |
| Aβ-/ND+ (SNAP)^*^ | 0.67 (0.28-1.46) | 0.341 |
| Aβ+/ND+^*^ | **12.12 (7.25-20.69)** | **<0.001** |

**Boldface** signifies effects that are significant at p<0.05.

**^*^** 4 level categorical variable for biomarker group with Aβ-/ND- set as the referent

Table I: Associations between Biomarker Groups and APOE Carrier Status When Adjusting for White Matter Hyperintensity Burden

|  | **Odds Ratio (95% CI)** | **p-value** |
| --- | --- | --- |
| ***APOE ε2*** |  |  |
| Age | 0.99 (0.94-1.04) | 0.723 |
| Sex (female) | 1.07 (0.60-1.90) | 0.813 |
| Cognitive Diagnosis (MCI) | 0.75 (0.40-1.38) | 0.356 |
| White Matter Hyperintensity Volume | 1.03 (0.98-1.06) | 0.181 |
| Aβ+/ND-^*^ | **0.52 (0.26-0.98)** | **0.049** |
| Aβ-/ND+ (SNAP)^*^ | 0.90 (0.28-2.39) | 0.838 |
| Aβ+/ND+^*^ | **0.21 (0.06-0.63)** | **0.010** |
| ***APOE ε4*** |  |  |
| Age | **0.90 (0.86-0.93)** | **<0.001** |
| Sex (female) | 0.98 (0.65-1.48) | 0.930 |
| Cognitive Diagnosis (MCI) | 1.19 (0.75-1.88) | 0.459 |
| White Matter Hyperintensity Volume | 0.99 (0.96-1.02) | 0.548 |
| Aβ+/ND-^*^ | **7.54 (4.61-12.63)** | **<0.001** |
| Aβ-/ND+ (SNAP)^*^ | 0.30 (0.05-1.08) | 0.113 |
| Aβ+/ND+^*^ | **14.67 (7.65-29.12)** | **<0.001** |

**Boldface** signifies effects that are significant at p<0.05.

**^*^** 4 level categorical variable for biomarker group with Aβ-/ND- set as the referent

Table J: Associations between Biomarker Groups and APOE Carrier Status When Defining Amyloid Positivity using PET Imaging and Neurodegeneration using only 3T Images

|  | **Odds Ratio (95% CI)** | **p-value** |
| --- | --- | --- |
| ***APOE ε2*** |  |  |
| Age | 1.00 (0.96-1.04) | 0.921 |
| Sex (female) | 1.03 (0.62-1.69) | 0.914 |
| Cognitive Diagnosis (MCI) | 0.79 (0.46-1.34) | 0.382 |
| Aβ+/ND-^*^ | 0.54 (0.27-1.01) | 0.061 |
| Aβ-/ND+ (SNAP)^*^ | 1.11 (0.48-2.36) | 0.799 |
| Aβ+/ND+^*^ | **0.40 (0.15-0.93)** | **0.043** |
| ***APOE ε4*** |  |  |
| Age | **0.92 (0.89-0.95)** | **<0.001** |
| Sex (female) | 0.89 (0.62-1.27) | 0.511 |
| Cognitive Diagnosis (MCI) | 1.24 (0.84-1.83) | 0.269 |
| Aβ+/ND-^*^ | 5.12 (3.36-7.88) | **<0.001** |
| Aβ-/ND+ (SNAP)^*^ | 0.71 (0.32-1.47) | 0.381 |
| Aβ+/ND+^*^ | **10.46 (6.08-18.46)** | **<0.001** |

**Boldface** signifies effects that are significant at p<0.05.

**^*^** 4 level categorical variable for biomarker group with Aβ-/ND- set as the referent

Table K: Association between Polygenic Risk Score for AD and Biomarker Groups

|  | **Beta (95% CI)** | **p-value** |
| --- | --- | --- |
| ***Neurodegeneration Definition*** |  |  |
| Age | 9x10^-6^ (-1x10^-4^ - 1x10^-4^) | 0.871 |
| Sex (female) | 9x10^-4^ (-5x10^-4^ - 2x10^-3^) | 0.204 |
| Cognitive Diagnosis (MCI) | 2x10^-3^ (-3x10^-5^ - 3x10^-3^) | 0.054 |
| Aβ+/ND-^*^ | 1x10^-3^ (-7x10^-4^ - 3x10^-3^) | 0.224 |
| Aβ-/ND+ (SNAP)^*^ | 6x10^-4^ (-2x10^-3^ - 3x10^-3^) | 0.637 |
| Aβ+/ND+^*^ | 1x10^-3^ (-6x10^-4^ - 3x10^-3^) | 0.167 |
| ***CSF Tau Definition*** |  |  |
| Age | 1x10^-5^ (-1x10^-4^ - 1x10^-4^) | 0.801 |
| Sex (female) | 7x10^-4^ (-7x10^-4^ - 2x10^-3^) | 0.344 |
| Cognitive Diagnosis (MCI) | **2x10^-3^ (3x10^-4^ - 3x10^-3^)** | **0.022** |
| Aβ+/Tau-^*^ | 1x10^-3^ (-7x10^-4^ - 3x10^-3^) | 0.258 |
| Aβ-/Tau+ (SNAP)^*^ | **4x10^-3^ (7x10^-4^ - 8x10^-3^)** | **0.018** |
| Aβ+/Tau+^*^ | **2x10^-3^ (8x10^-5^ - 4x10^-3^)** | **0.041** |

**Boldface** signifies effects that are significant at p<0.05.

**^*^** 4 level categorical variable for biomarker group with Aβ-/ND- set as the referent
